# RTKN2 Inhibits the Growth, Migration, Invasion and Glycolysis of Lung Adenocarcinoma Cells by Inactivating the NF-κB Signalling Pathway

**DOI:** 10.1007/s10528-023-10352-6

**Published:** 2023-03-23

**Authors:** Na Wang, Jinxiang Wang

**Affiliations:** Department of Respiratory and Critical Care Medicine, Beijing Luhe Hospital, Capital Medical University, Xinhuananlu No. 82, Tongzhou District, Beijing, 101101 China

**Keywords:** Lung adenocarcinoma, RTKN2, NF-Κb, p65, Glycolysis

## Abstract

**Background:**

Lung adenocarcinoma (LUAD) is a malignant tumour that seriously threatens the life and health of people worldwide. This research was carried out to investigate the role of Rhotekin 2 (RTKN2) in LUAD progression.

**Methods and Results:**

The GEPIA online database was used to analyse abnormally expressed genes in lung adenocarcinoma and RTKN2 expression in various cancers. Cell proliferation was detected with CCK-8 and colony formation assays. Transwell assays were carried out to assess cell migration and invasion. The extracellular acidification rate (ECAR) and oxygen consumption rate (OCR) were evaluated by a Seahorse XFe96 analyser. The interaction between RTKN2 and p65 was confirmed using a coimmunoprecipitation assay. RTKN2 expression was detected with qPCR, immunohistochemistry, and western blot assays. The p65 levels in the cytoplasm and nucleus were determined by western blot assays. RTKN2 levels were prominently decreased in LUAD tissues and cell lines. RTKN2 overexpression suppressed LUAD cell growth, invasion, migration, and glycolysis, while RTKN2 knockdown showed the opposite effects. Additionally, p65 could be negatively regulated by RTKN2. RTKN2 overexpression increased p65 levels in the cytoplasm but decreased p65 levels in the nucleus. Furthermore, blocking the NF-κB signalling pathway neutralized the effect of RTKN2 silencing in LUAD cells.

**Conclusion:**

RTKN2 inhibited the malignant behaviour and glycolysis of LUAD cells by blocking the NF-κB signalling pathway, implying that RTKN2 could be a cancer suppressor in LUAD progression.

**Supplementary Information:**

The online version contains supplementary material available at 10.1007/s10528-023-10352-6.

## Introduction

Lung cancer is a common malignant neoplasm in China that seriously threatens the life and health of people. Eighty-five per cent of lung cancer cases are non-small cell lung cancer (NSCLC), of which lung adenocarcinoma (LUAD) is the most common subtype (Herbst et al. 2018; Jonna S and Subramaniam 2019). Over the past decade, rapid developments have been made in surgical removal, local ablation, vascular intervention and molecular targeted therapy for the treatment of lung cancer; however, the 5-year survival rate remains low (Ettinger et al. 2017; Hu et al. 2020). Hence, elucidation of the molecular mechanism of LUAD and identification of novel therapeutic targets are urgently needed.

The nuclear transcription factor κB (NF-κB) signalling pathway was confirmed to participate in the regulation of biological behaviours, including cell proliferation and apoptosis (Lawrence 2009; Peng et al. 2020). After cytotoxic stimulation, IκB, which forms a complex with NF-κB, is phosphorylated and degraded by IκB kinase. Then, free active NF-κB (p65) translocates to the nucleus to regulate the transcriptional activity of antiapoptotic proteins (Bernal et al. 2006). Previous studies have demonstrated that NF-κB participates in the proliferation of lung cancer cells (Yu et al. 2017; Guo et al. 2020). Activated NF-κB mainly promotes the transformation into S phase and M phase of the lung cancer cell cycle by modulating the expression of proliferation-related proteins (Yang et al. 2015; Xu et al. 2013). Additionally, NF-κB mainly acts as an inhibitor of apoptosis in lung cancer (Zhang et al. 2012). However, under certain circumstances, NF-κB can also induce apoptosis of lung cancer cells (Wang et al. 2013). The specific mechanism underlying the progression of lung cancer is still unclear. Thus, further research is needed to provide a better understanding of lung cancer progression.

Rhotekin (RTKN) is a small GTP binding protein that can regulate cell growth, differentiation and apoptosis by binding with activated Rho molecules (Collier et al. 2004). The RTKN proteins are divided into RTKN 1 and 2, which have the same Rho-GTPase-binding domain (Ito et al. 2018). Many studies have demonstrated that RTKN is abnormally expressed in various malignant tumours and closely related to tumour cell invasion, metastasis and poor prognosis of patients (Pang et al. 2017; Li et al. 2016).

Glucose metabolism is the main way for cells to obtain energy (Mulukutla et al. 2016). Recently, metabolic reprogramming based on aerobic glycolysis has become widespread in cancer cells to provide energy for cancer cell growth and aggravate the proliferation and metastasis of malignant tumours (Abdel-Wahab et al. 2019; Vander et al. 2009). However, to date, whether RTKN2 participates in the regulation of glycolysis in LUAD is still unknown.

Here, the present research determined that RTKN2 was expressed at low levels in LUAD. We aimed to further investigate the function and precise mechanism of RTKN2 in LUAD development.

## Materials and Methods

### Bioinformatic Analysis

The differentially expressed genes on each chromosome of lung cancer and RTKN2 expression in cancers were predicted by the GEPIA online database (http://gepia.cancer-pku.cn/).

### Tissue Samples

Research involving human tissues was approved by the ethics committee of Beijing Luhe Hospital, and LUAD tissues and paracarcinoma lung (para-LUAD) tissues were obtained from patients with lung cancer during surgery (*n* = 59). All tissues were stored at − 80 °C until further use. Written informed consent was obtained from all subjects.

### Immunohistochemical (IHC) Analysis

LUAD and para-LUAD tissues were embedded in paraffin and cut into 5 μm sections. Tissue sections were deparaffinized and rehydrated. Antigen retrieval was performed using 0.01 M citric acid buffer. After PBS washes, the sections were blocked using normal goat serum. The sections were incubated with anti-RTKN2 at 4 °C overnight and incubated with secondary antibody at 37 °C for 30 min. After staining with DAB solution, the results were observed and photographed under a microscope (Olympus, Tokyo, Japan).

### Cell Culture and Transfection

LUAD cell lines, including PC9, A549, MES-1, H460 and H1299, and the normal lung cell line 16HBE were acquired from ATCC (USA). All cells were cultured with DMEM (HyClone) with 10% FBS (HyClone) at 37 °C in a 5% CO2 atmosphere. After 24 h of incubation, the cells were transfected with the pcDNA3.1 vector to construct the RTKN2 overexpression vector and empty vector. The cells were transfected with short hairpin RNA of RTKN2 (sh-RTKN2) and short hairpin RNA negative control (sh-nc) to knock down RTKN2 expression. All vectors and shRNAs were synthesized and purchased from Guangzhou RiboBio, and transfection was carried out using Lipofectamine 2000 (Invitrogen). For inhibition of the activation of the NF-κB signalling pathway, 0.6 nM bortezomib was used to treat the cells for 24 h.

### Quantitative Real-Time Polymerase Chain Reaction (qPCR)

Total RNA was extracted using a total RNA extraction kit (Solarbio). A BeyoRT™ II cDNA synthesis kit (Beyotime) was utilized to synthesize cDNA using 1 μg RNA. qPCR was conducted using the SYBR Green Master Mix kit (TaKaRa) on an ABI 7500 real-time system. The reaction conditions were as follows: 95 °C predenaturation for 1 min, 95 °C for 15 s, 60 °C for 15 s, 45 cycles. The relative expression of RTKN2 was calculated using the 2-ΔΔCt method and normalized to GAPDH. The primer sequences were as follows (5'—> 3').

RTKN2: Forward Primer, ATGCTCGACTAATGGCCTATACA; Reverse Primer, CGTCGTGATCGTTCTTTATTGCT.

GAPDH: Forward Primer, TGTGGGCATCAATGGATTTGG; Reverse Primer, ACACCATGTATTCCGGGTCAAT.

### Cell Viability Assay

A Cell Counting Kit-8 (CK04, Dojindo, Kumamoto, Japan) was utilized to determine cell viability. All transfected cells were seeded into 96-well plates at a density of 5 × 10^3^ cells/well and maintained at 37 °C for 24 h. Subsequently, 10 μL of CCK-8 solution was incubated with the cells for 2 h. Absorbance was detected using a microplate reader (Bio-Rad, Hercules, CA) at 450 nm.

### Flow Cytometry

Flow cytometry was used to detect the apoptosis rate of LUAD cells in each group. Cells were seeded into 96-well plates (2 × 10^4^ cells/well) and cultured until the cell density reached 80%. According to the Annexin V-APC/7-AAD kit (Sigma-Aldrich) instructions, flow cytometry was used to detect the apoptosis rate in a BDVerse flow cytometry system (BD, USA).

### Colony Formation Detection

All transfected cells were plated in six-well plates at a density of 1000 cells/well and cultured at 37 °C with 5% CO2. After 2 weeks, cells were immobilized with 4% paraformaldehyde (PFA) and stained with crystal violet. The stained cells were observed under a microscope (Olympus).

### Cell Migration and Invasion Analysis

Chambers without Matrigel and Matrigel-coated chambers (BD Biosciences, San Jose, CA) were used for cell migration and invasion detection, respectively. Transfected cells were seeded in the upper chambers, and medium was added to the lower chambers. After 24 h, the migrated and invaded cells were immobilized with 4% paraformaldehyde and stained with crystal violet. The results were visualized under a microscope (Olympus). The number of migrated and invaded cells was quantified in 5 random fields.

### Measurement of Glycolysis

The oxygen consumption rate (OCR) and extracellular acidification rate (ECAR) were measured as previously described (Lai et al. 2020). Briefly, A549 and H1299 cells were seeded and cultured in 96-well plates. For OCR detection, the Seahorse automatically filled with oligomycin at 20 min, p-trifluoromethoxy carbonyl cyanide phenylhydrazone (FCCP) at 50 min, and antimycin A & rotenone at 80 min. For ECAR detection, glucose was added at 20 min, oligomycin was added at 50 min, and 2-deoxy-D-glucose (2-DG) was added at 80 min using Seahorse auto fill. OCR was detected using a MitoCheck Mitochondrial OCR assay kit (MX-200-4, Agilent, Beijing). ECAR was detected using a Seahorse XF glycolysis stress test kit (103020–100, Agilent, Beijing) from 0 to 110 min following the manufacturer’s protocol. The results were measured using a Seahorse XF96 Analyzer (Agilent).

### Western Blot

Proteins were extracted from cells using RIPA buffer. After the protein concentration was detected using a BCA kit (Sigma), 20 µg of protein was separated by 10% SDS‒PAGE and then transferred to PVDF membranes. Then, the membranes were blocked with 5% skim milk for 2 h, incubated with primary antibodies (anti-p65, ab32536; anti-RTKN2, ab154954; anti-AKT, ab8805; anti-p-AKT, ab38449; anti-p38, ab170099; anti-p-p38, ab211061; anti-β-catenin, ab32572; anti-IκB, ab32518; anti-p-IκB, ab133462; anti-GAPDH, ab8245; anti-H3, ab1791) at 4 °C overnight, followed by incubation with the secondary antibody (Abcam) at 25 °C for 1 h. An ECL kit (Abcam) was used for visualization of protein bands. Grey value analysis was performed and normalized to GAPDH.

### Coimmunoprecipitation (co-IP) Assay

According to a previous study (Zhou et al. 2021), RTKN2-transfected cells were lysed using lysis buffer and centrifuged at 12,000 × g for 10 min. The supernatant was collected and incubated with Protein A/G PLUS-Agarose (Santa Cruz, Santa Cruz, CA) along with anti-p65 at 4 °C overnight. Following washing, the immunoprecipitants were boiled with 2 × SDS Loading Buffer. The expression of p65 was measured using western blotting as mentioned above.

### RNA Fluorescence in Situ Hybridization (FISH)

Cy3-labelled p65 probes were designed and synthesized by Sangon (Shanghai). A FISH kit (GenePharma) was used to perform the FISH assay. Briefly, A549 and H1299 cells were fixed with ethanol for 15 min and treated with Triton X-100 for 15 min. The cells were hybridized with denatured probe buffer at 37 °C overnight. After washes with SSC solution, the cells were incubated with DAPI for 20 min. The signals were observed and photographed under a confocal laser scanning microscope.

### Statistical Analysis

Statistics were analysed using GraphPad Prism 7 software. All data are shown as the mean±SD. Differences were compared by Student’s t test (two groups) or one-way ANOVA (three or more groups). *P* < 0.05 was regarded as statistically significant.

## Results

### RTKN2 was Downregulated in LUAD

**Fig. 1:**
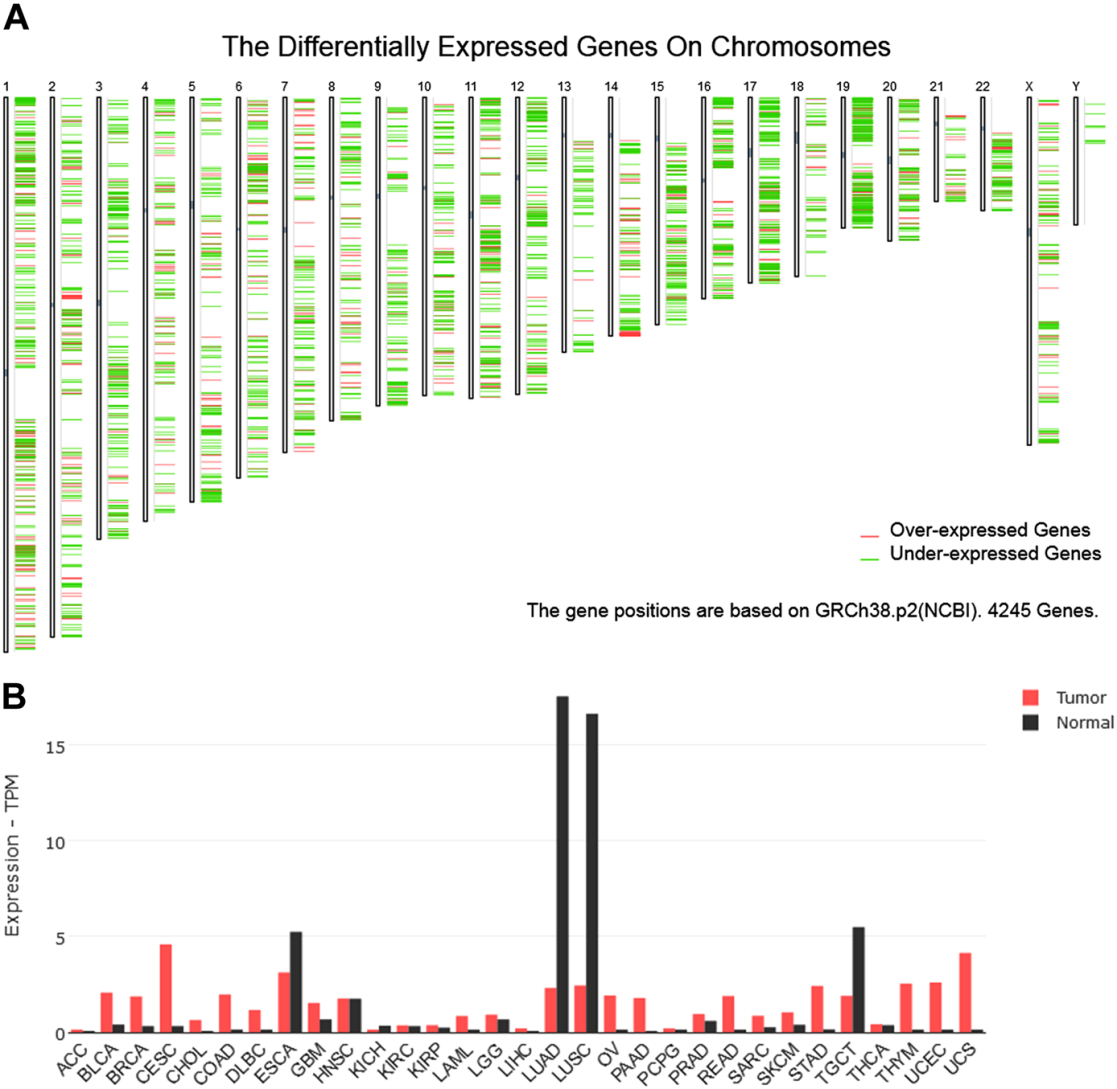
RTKN2 was downregulated in LUAD. The dysregulated genes in LUAD **A** and the RTKN2 levels in cancers **B** were analysed using the GEPIA database

To explore differentially expressed genes in LUAD, we searched the GEPIA database. As displayed in Fig. 1A, there were multiple highly expressed and lowly expressed genes on each chromosome in LUAD, and RTKN2 was one of the downregulated genes. The gene names and expressions were showed in supplementary table 1. Additionally, the GEPIA database results showed that RTKN2 was expressed at low levels in LUAD as well as LUSC (Fig. 1B).

### RTKN2 was Decreased in LUAD Tissues as Well as Cells

**Fig. 2:**
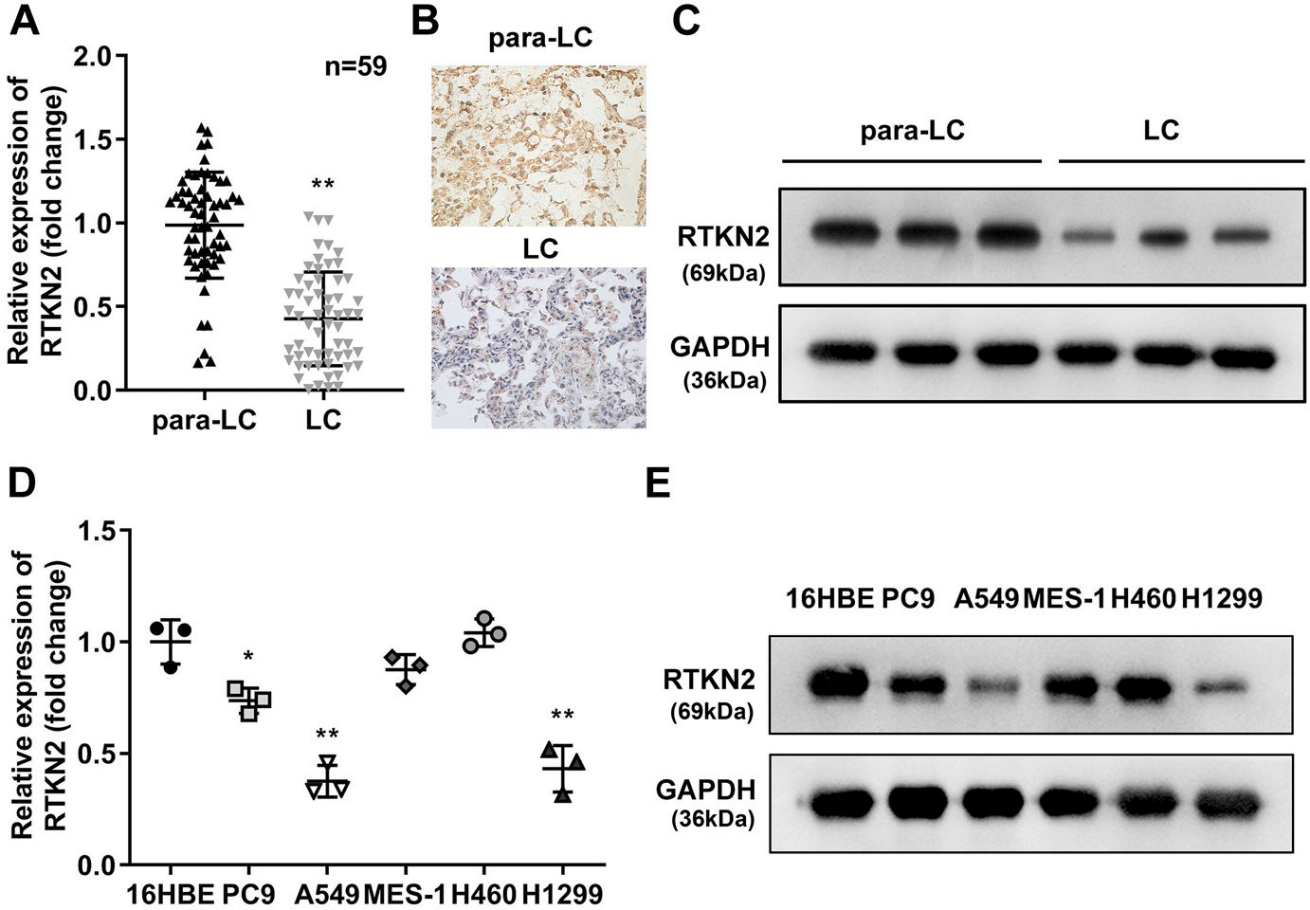
RTKN2 was downregulated in LUAD tissues and cells. The RTKN2 levels in LUAD tissues were analysed by qRT‒PCR **A**, immunohistochemistry **B** and western blotting **C** The RTKN2 levels in LUAD cells were analysed by qRT‒PCR **D** and western blotting **E**. **P < 0.01

To further confirm whether RTKN2 is expressed at low levels in LUAD, we collected 59 paired LUAD tissues and para-LUAD tissues for further qPCR detection. The results illustrated that the mRNA levels of RTKN2 were dramatically lower in the LUAD tissues than in the para-LUAD tissues (Fig. 2A), which was further confirmed by the results of IHC (Fig. 2B). Additionally, the western blot results showed that the protein levels of RTKN2 were dramatically lower in the LUAD tissues than in the para-LUAD tissues (Fig. 2C). Furthermore, we confirmed that RTKN2 levels were prominently lower in PC9, A549 and H1299 cells at both the mRNA (Fig. 2D) and protein (Fig. 2E) levels than in 16HBE cells (Fig. 2D-E).

### RTKN2 Overexpression Suppressed Proliferation, Migration, Invasion and Glycolysis of LUAD Cells

**Fig. 3:**
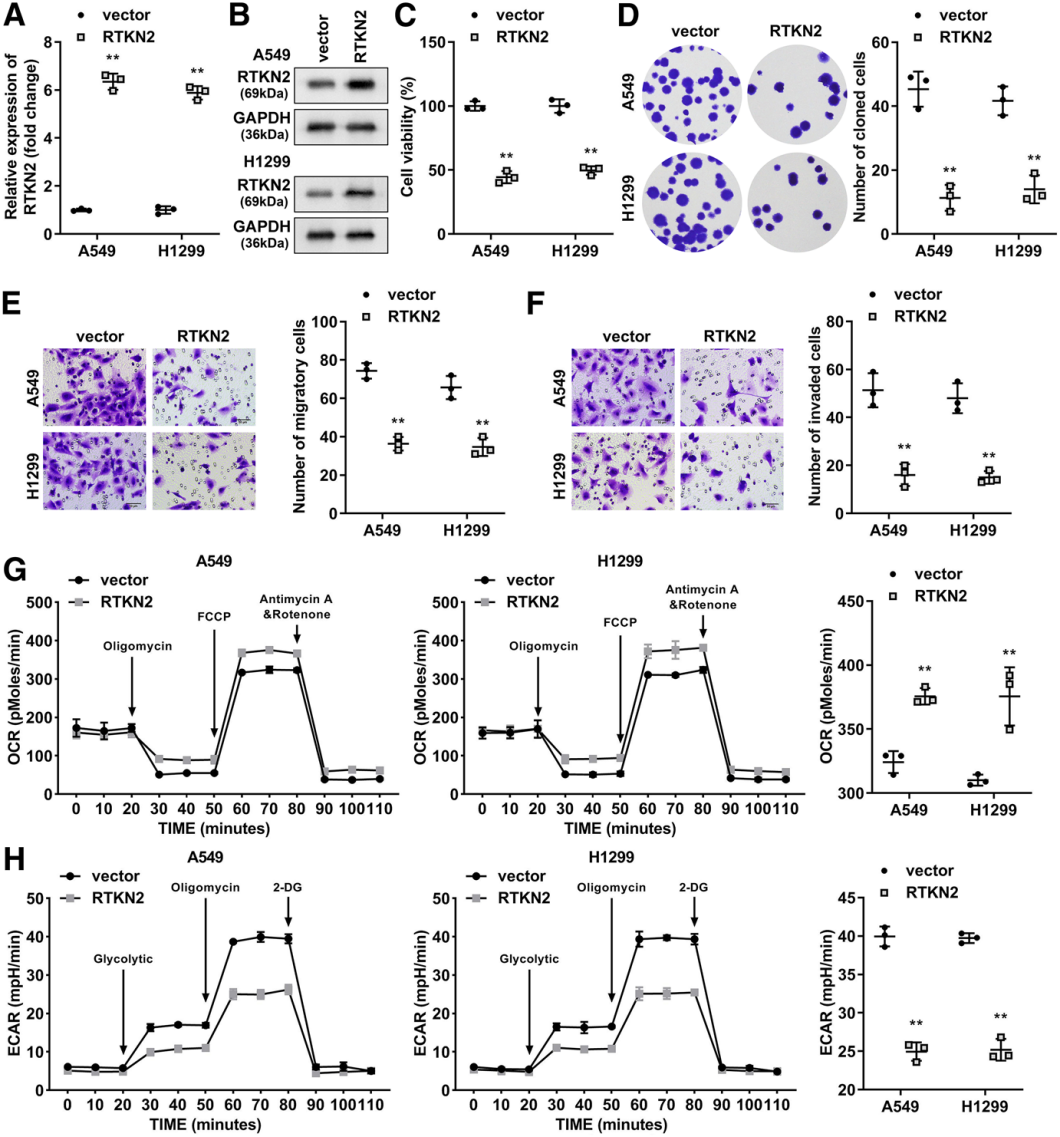
RTKN2 overexpression suppresses cell proliferation, migration, invasion, and glycolysis in LUAD cells. **A** RTKN2 expression was detected using qPCR and **B** western blot in RTKN2-overexpressing vector-transfected cells. Cell proliferation was evaluated by detecting cell viability using CCK-8 assays **C** and cell colonies using colony formation assay **D**. Transwell assays were used to measure cell migration **E** and cell invasion **F**. The OCR **G** and ECAR **H** were analysed as indicators of glycolysis in tumour cells. **P < 0.01

To investigate the biological functions of RTKN2, we first transfected A549 and H1299 cells with the RTKN2 overexpression vector or empty vector. RTKN2 was markedly upregulated in RTKN2-overexpressing vector-transfected cells at mRNA levels (Fig. 3A) and protein levels (Fig. 3B). The overexpression of RTKN2 prominently inhibited cell viability and colony formation (Fig. 3C and D). Subsequently, RTKN2 overexpression prominently inhibited cell migration and invasion (Fig. 3E and F). Moreover, to explore the regulatory effect of RTKN2 on the status of aerobic and anaerobic metabolism, we analysed the extracellular acidification rate (ECAR) and oxygen consumption rate (OCR). RTKN2 prominently increased the OCR (Fig. 3G) but decreased the ECAR (Fig. 3H) in A549 and H1299 cells. Specifically, the steady-state glycolytic flux and glycolytic capacity in the RTKN2 overexpression group were decreased, and basal respiration and ATP production were increased, suggesting that RTKN2 inhibited glycolytic development.

### RTKN2 Directly Interacts with NF-κB

**Fig. 4:**
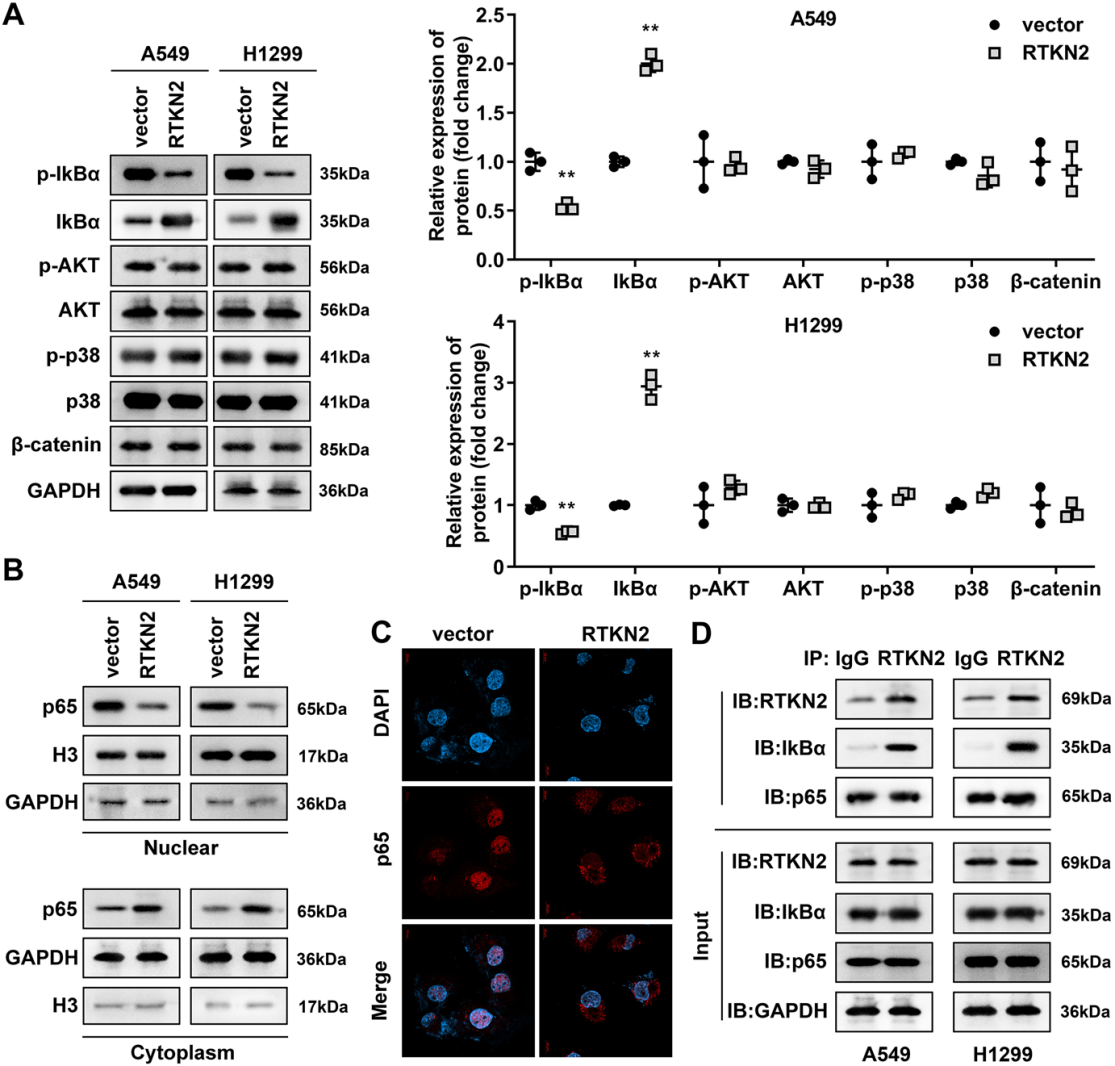
RTKN2 directly acted on the NF-κB signalling pathway. **A** The p-AKT, p38, β-catenin and p-IκBα levels in LUAD cells were determined by western blot assays. **B** The p65 protein levels in LUAD cells were determined by western blotting. **C** RTNK2 and p65 expression were analysed with FISH. **D** The combination of RTKN2 and P65 was confirmed using a co-IP assays. **P < 0.01

Then, we analysed the effects of PTKN2 on cell proliferation and immune-related pathways. Interestingly, we found that RTKN2 overexpression dramatically decreased the phosphorylation of IκBα and showed no effects on the phosphorylation levels of AKT, p38 and β-catenin (Fig. 4A). Then, we found that RTKN2 overexpression dramatically decreased the p65 protein levels in the nucleus and increased them in the cytoplasm (Fig. 4B). The FISH results showed that RTKN2 overexpression inhibited the nuclear translocation of p65 (Fig. 4C). In addition, the combination of RTKN2 and P65/IκB was confirmed with Co-IP assays. We found that RTKN2 bound to p65 and IκB (Fig. 4D).

### Bortezomib Treatment Neutralized the Effect of RTKN2 Silencing in LUAD Cells

**Fig. 5:**
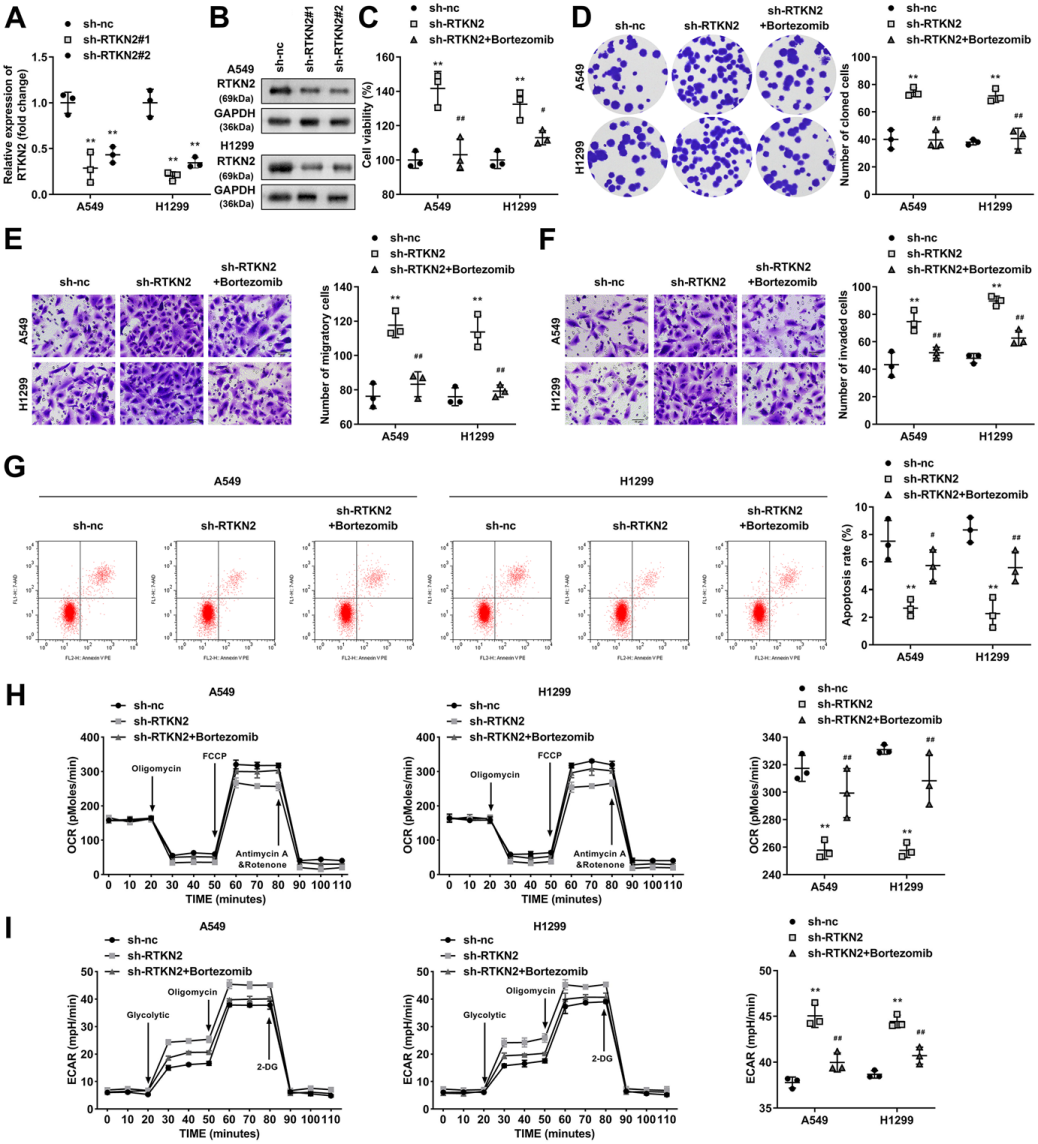
Bortezomib treatment neutralized the effect of sh-RTKN2 in LUAD cells. RTKN2 expression was detected using qPCR **A** and western blot **B** in sh-RTKN2-transfected cells. Cell proliferation was evaluated by detecting cell viability using CCK-8 assays **C** and cell colonies using colony formation assays **D**. Transwell assays were used to measure cell migration **E** and cell invasion **F**. **G** Flow cytometry was conducted to detect the cell apoptosis rate. The OCR **H** and ECAR **I** were analysed as indicators of glycolysis in tumour cells. **P < 0.01. ##P < 0.01

Bortezomib is an effective protease inhibitor that was used to inhibit the NF-κB signalling pathway in this study. RTKN2 was markedly downregulated in the sh-RTKN2-transfected cells at mRNA (Fig. 5A) and protein levels (Fig. 5B), and sh-RTKN2 #1 was selected for next experiments. The downregulation of RTKN2 prominently promoted the viability, colony formation, migration and invasion of LUAD cells (Fig. 5C-F). Besides, the apoptosis rate of LUAD cells was decreased after RTKN2 knockdown (Fig. 5G). Moreover, RTKN2 knockdown prominently reduced the OCR and enhanced the ECAR in A549 and H1299 cells (Fig. 3H and I). Specifically, the steady-state glycolytic flux and glycolytic capacity in the RTKN2 knockdown group were increased, and basal respiration and ATP production were decreased. After bortezomib treatment, the effects of sh-RTKN2 on cell viability, colony formation, migration, invasion, apoptosis and glycolysis were reversed, implying that the NF-κB signalling pathway mediates RTKN2 function in LUAD.

## Discussion

RTKN2, a newly discovered Rho-GTPase effector protein, was first found to be a scaffold protein acting with the GTP-bound form of Rho (Reid et al. 1996). Abnormally expressed RTKN2 was demonstrated to modulate the development of various cancers. For example, Wang et al. (Wang X et al. 2018) demonstrated that RTKN2 knockdown promoted G1 phase arrest and apoptosis in human osteosarcoma, implying that RTKN2 acted as an oncogene in human osteosarcoma cell survival. However, Guo et al. (Guo et al. 2021) confirmed that RTKN2, as an independent risk factor for the poor prognosis of LUAD patients, was downregulated in lung cancer cells. Additionally, the different effects may be caused by different targets of RTKN2. In this study, bioinformatic analysis indicated that RTKN2 was expressed at low levels in lung cancer cells, which was further confirmed by qPCR and western blot assays. Functional studies revealed that RTKN2 overexpression suppressed cell growth and metastasis in LUAD cells.

The main metabolic characteristic of cancer cells is aerobic glycolysis. Even under oxygen-enriched conditions, cancer cells rely on glycolysis to consume glucose and produce large amounts of lactic acid. This phenomenon greatly accelerates tumour growth (Kayawake et al. 2019; Lee et al. 2019; Li et al. 2019). Targeting the glycolytic metabolic pathway might be an effective way to control tumour growth and enhance anticancer efficacy in LUAD. In the present study, we found that RTKN2 suppressed glycolysis in LUAD cells but not proliferation, migration or invasion. These results indicated the inhibitory role of RTKN2 in LUAD progression.

NF-κB is a family of mammalian transcription factors and plays an important role in the progression of many human diseases (Baldwin 2001). Previous studies have demonstrated that NF-κB affects the differentiation and proliferation of various cells. Activated NF-κB participates in the regulation of apoptosis, expression of angiogenic proteins and carcinogenesis (Fan et al. 2013; Patel et al. 2018). P65 is the most common member of the NF-κB family and is quickly activated by various stimuli (Valovka and Hottiger 2011). NF-κB activity is inhibited by combining with inhibitory proteins such as IκB to form inactive complexes. The phosphorylation of IκB activates NF-κB. Activated NF-κB enters the nucleus and contacts DNA to regulate the transcription of downstream genes (Bacher et al. 2021). In this study, we demonstrated that RTKN2 could directly bind to p65. Additionally, western blot and FISH assays showed that RTKN2 could prevent NF-κB from entering the nucleus. Mechanistically, RTKN2 binds to p65, thus, inhibiting its nuclear translocation, resulting in the upregulation of p65 protein in the cytoplasm and its downregulation in the nucleus. Overexpression of RTKN2 reduced the phosphorylation of IκB α. Additionally, after treatment with bortezomib (an effective protease inhibitor used to inhibit the NF-κB signalling pathway), the effect of RTKN2 knockdown in LUAD cells was neutralized. These results also suggested that RTKN2 inhibited glycolysis development in LUAD by inhibiting the nuclear translocation of p65, which further blocked the NF-κB signalling pathway. We speculated that the specific mechanism by which the NF-κB signalling pathway regulates glycolysis may be through regulating the expression of some key glycolytic enzymes. However, this study explored the relationship between the NF-κB signalling pathway and glycolysis in LUAD for the first time. In the future, we will carry out more experiments to explore the relationship between the NF-κB signalling pathway and glycolysis. Additionally, an animal study was not performed here, which could be a limitation of this study.

In summary, our research found that RTKN2 prevented the growth and metastasis of LUAD cells by suppressing glycolysis and the NF-κB signalling pathway.

### Supplementary Information

Below is the link to the electronic supplementary material.

Supplementary file1 (TXT 448 KB)

## Data Availability

The datasets used and analysed during the current study are available from the corresponding author on reasonable request.

## References

[CR1] Abdel-Wahab AF, Mahmoud W, Al-Harizy RM (2019). Targeting glucose metabolism to suppress cancer progression: prospective of anti-glycolytic cancer therapy. Pharmacol Res.

[CR2] Bacher S, Meier-SoeLUADh J, Kracht M, Schmitz ML (2021). Regulation of Transcription Factor NF-kappaB in Its Natural Habitat: The Nucleus. Cells-Basel.

[CR3] Baldwin AJ (2001). Series introduction: the transcription factor NF-kappaB and human disease. J Clin Invest.

[CR4] Bernal-Mizrachi L, Lovly CM, Ratner L (2006). The role of NF-{kappa}B-1 and NF-{kappa}B-2-mediated resistance to apoptosis in lymphomas. Proc Natl Acad Sci USA.

[CR5] Collier FM, Gregorio-King CC, Gough TJ, Talbot CD, Walder K, Kirkland MA (2004). Identification and characterization of a lymphocytic Rho-GTPase effector: rhotekin-2. Biochem Biophys Res Commun.

[CR6] Ettinger DS, Wood DE, Aisner DL, Akerley W, Bauman J, Chirieac LR, D'Amico TA, DeCamp MM, Dilling TJ, Dobelbower M, Doebele RC, Govindan R, Gubens MA, Hennon M, Horn L, Komaki R, Lackner RP, Lanuti M, Leal TA, Leisch LJ, Lilenbaum R, Lin J, Loo BJ, Martins R, Otterson GA, Reckamp K, Riely GJ, Schild SE, Shapiro TA, Stevenson J, Swanson SJ, Tauer K, Yang SC, Gregory K, Hughes M (2017). Non-small cell lung cancer, version 5.2017, NCCN clinical practice guidelines in oncology. J Natl Compr Canc Netw.

[CR7] Fan Y, Mao R, Yang J (2013). NF-kappaB and STAT3 signaling pathways collaboratively link inflammation to cancer. Protein Cell.

[CR8] Guo Q, Li D, Luo X, Yuan Y, Li T, Liu H, Wang X (2021). The regulatory network and potential role of LINC00973-miRNA-mRNA ceRNA in the progression of non-small-cell lung cancer. Front Immunol.

[CR9] Guo S, Wang Y, Li Y, Li Y, Feng C, Li Z (2020). Daidzein-rich isoflavones aglycone inhibits lung cancer growth through inhibition of NF-kappaB signaling pathway. Immunol Lett.

[CR10] Herbst RS, Morgensztern D, Boshoff C (2018). The biology and management of non-small cell lung cancer. Nature.

[CR11] Hu D, Li S, Huang Z, Wu N, Lu X (2020). Predicting postoperative non-small cell lung cancer prognosis via long short-term relational regularization. Artif Intell Med.

[CR12] Ito H, Morishita R, Nagata KI (2018). Functions of Rhotekin, an Effector of Rho GTPase, and Its Binding Partners in Mammals. Int J Mol Sci.

[CR13] Jonna S, Subramaniam DS (2019). Molecular diagnostics and targeted therapies in non-small cell lung cancer (NSCLUAD): an update. Discov Med.

[CR14] Kayawake H, Okumura N, Yamanashi K, Takahashi A, Itasaka S, Yoshioka H, Nakashima T, Matsuoka T (2019). Non-small cell lung cancer with pathological complete response: predictive factors and surgical outcomes. Gen Thorac Cardiovasc Surg.

[CR15] Lai Z, Wei T, Li Q, Wang X, Zhang Y, Zhang S (2020). Exosomal circFBLIM1 Promotes Hepatocellular Carcinoma Progression and Glycolysis by Regulating the miR-338/LRP6 Axis. Cancer Biother Radiopharm.

[CR16] Lawrence T (2009). The nuclear factor NF-kappaB pathway in inflammation. CSH Perspect Biol.

[CR17] Lee SJ, Yoo JW, Ju S, Cho YJ, Kim JD, Kim SH, Jang IS, Jeong BK, Lee GW, Jeong YY, Kim HC, Bae K, Jeon KN, Lee JD (2019). Quantitative severity of pulmonary emphysema as a prognostic factor for recurrence in patients with surgically resected non-small cell lung cancer. Thorac Cancer.

[CR18] Li B, Chen P, Chang Y, Qi J, Fu H, Guo H (2016). Let-7a inhibits tumor cell growth and metastasis by directly targeting RTKN in human colon cancer. Biochem Bioph Res Co.

[CR19] Li Y, Xu Q, Yang W, Wu T, Lu X (2019). Oleanolic acid reduces aerobic glycolysis-associated proliferation by inhibiting yes-associated protein in gastric cancer cells. Gene.

[CR20] Mulukutla BC, Yongky A, Le T, Mashek DG, Hu WS (2016). Regulation of glucose metabolism—a perspective from cell bioprocessing. Trends Biotechnol.

[CR21] Pang X, Li R, Shi D, Pan X, Ma C, Zhang G, Mu C, Chen W (2017). Knockdown of Rhotekin 2 expression suppresses proliferation and induces apoptosis in colon cancer cells. Oncol Lett.

[CR22] Patel M, Horgan PG, McMillan DC, Edwards J (2018). NF-κB pathways in the development and progression of colorectal cancer. Transl Res.

[CR23] Peng C, Ouyang Y, Lu N, Li N (2020). The NF-κB Signaling pathway, the microbiota, and gastrointestinal tumorigenesis: recent advances. Front Immunol.

[CR24] Reid T, Furuyashiki T, Ishizaki T, Watanabe G, Watanabe N, Fujisawa K, Morii N, Madaule P, Narumiya S (1996). Rhotekin, a new putative target for Rho bearing homology to a serine/threonine kinase, PKN, and rhophilin in the rho-binding domain. J Biol Chem.

[CR25] Valovka T, Hottiger MO (2011). p65 controls NF-kappaB activity by regulating cellular localization of IkappaBbeta. Biochem J.

[CR26] Vander HM, Cantley LUAD, Thompson CB (2009). Understanding the Warburg effect: the metabolic requirements of cell proliferation. Science.

[CR27] Wang X, Zhang L, Wang W, Wang Y, Chen Y, Xie R, Li X, Wang Y (2018). Biosci Rep.

[CR28] Wang Y, Yue B, Yu X, Wang Z, Wang M (2013). SLUG is activated by nuclear factor kappa B and confers human alveolar epithelial A549 cells resistance to tumor necrosis factor-alpha-induced apoptosis. World J Surg Oncol.

[CR29] Xu TP, Shen H, Liu LX, Shu YQ (2013). Plumbagin from *Plumbago Zeylanica* L induces apoptosis in human non-small cell lung cancer cell lines through NF- kappaB inactivation. Asian Pac J Cancer Prev.

[CR30] Yang L, Zhou Y, Li Y, Zhou J, Wu Y, Cui Y, Yang G, Hong Y (2015). Mutations of p53 and KRAS activate NF-kappaB to promote chemoresistance and tumorigenesis via dysregulation of cell cycle and suppression of apoptosis in lung cancer cells. Cancer Lett.

[CR31] Yu M, Qi B, Xiaoxiang W, Xu J, Liu X (2017). Baicalein increases cisplatin sensitivity of A549 lung adenocarcinoma cells via PI3K/Akt/NF-kappaB pathway. Biomed Pharmacother.

[CR32] Zhang L, Ruan J, Yan L, Li W, Wu Y, Tao L, Zhang F, Zheng S, Wang A, Lu Y (2012). Xanthatin induces cell cycle arrest at G2/M checkpoint and apoptosis via disrupting NF-kappaB pathway in A549 non-small-cell lung cancer cells. Molecules.

[CR33] Zhou L, Song Z, Hu J, Liu L, Hou Y, Zhang X, Yang X, Chen K (2021). ACSS3 represses prostate cancer progression through downregulating lipid droplet-associated protein PLIN3. Theranostics.

